# Relationship Between Maternal Iron Indices in the Second Trimester with Cord Blood Iron Indices and Pregnancy Outcomes: A Prospective Cohort Study

**DOI:** 10.3390/nu17091584

**Published:** 2025-05-05

**Authors:** J. P. Akshaykirthan, Manjunath S. Somannavar, M. S. Deepthy, Umesh Charantimath, S. Yogeshkumar, Amaresh Patil, Mrutyunjaya B. Bellad, Richard Derman, Shivaprasad S. Goudar

**Affiliations:** 1 Department of Biochemistry, Jawaharlal Nehru Medical College, KLE Academy of Higher Education and Research, Belagavi 590010, Karnataka, India; akshay.kirthan.jp@gmail.com; 2 Women’s & Children’s Health Research Unit, Jawaharlal Nehru Medical College, KLE Academy of Higher Education and Research, Belagavi 590010, Karnataka, India; deepthyms27@gmail.com (M.S.D.); amareshpatilp@jnmc.edu (A.P.); 3 Department of Community Medicine, Jawaharlal Nehru Medical College, KLE Academy of Higher Education and Research, Belagavi 590010, Karnataka, India; drumesh.charantimath@gmail.com (U.C.); yogeshkumar@jnmc.edu (S.Y.); 4 Department of Obstetrics and Gynecology, Jawaharlal Nehru Medical College, KLE Academy of Higher Education and Research, Belagavi 590010, Karnataka, India; belladmb@gmail.com; 5 Department of Global Affairs, Thomas Jefferson University, Philadelphia, PA 19107, USA; richard.derman@jefferson.edu; 6 Department of Physiology, Jawaharlal Nehru Medical College, KLE Academy of Higher Education and Research, Belagavi 590010, Karnataka, India; sgoudar@jnmc.edu

**Keywords:** iron deficiency anemia, pregnancy, maternal iron indices, neonatal outcomes, soluble transferrin receptor

## Abstract

**Background/Objectives:** Iron deficiency anemia in pregnancy poses risks to mothers and infants. This study aimed to correlate maternal iron indices in the second trimester with cord blood indices and pregnancy outcomes. **Methods:** This prospective cohort study was nested within the RAPIDIRON Trial (Reducing Anaemia in Pregnancy in India) at Jawaharlal Nehru Medical College, Karnataka, India. A total of 292 pregnant women with moderate anemia who received oral iron supplementation were enrolled from April 2021 to May 2023. Maternal iron indices were measured at multiple time points and correlated with cord blood indices and pregnancy outcomes. **Results:** Increased hemoglobin levels were observed in mothers of preterm and term neonates from 8.92 ± 0.81 vs. 9.02 ± 0.77 g/dL at 12–16 weeks to 11.14 ± 1.31 vs. 10.73 ± 1.24 g/dL at 26–30 weeks. A similar trend was observed in mothers across birth weight groups. Ferritin and TSAT levels significantly increased in all outcome groups (*p* < 0.001), peaking at 20–24 weeks and then slightly declining at 26–30 weeks. Additionally, maternal sTfR levels significantly improved from the early (7.72 ± 1.33 vs. 7.51 ± 1.61) to late second trimester (5.87 ± 0.81 vs. 5.76 ± 1.11) in mothers of both anemic and non-anemic neonates (*p* < 0.001). Maternal sTfR in other outcome groups also showed a similar pattern. A negligible correlation was found between maternal and cord blood iron indices. **Conclusions:** Maternal iron indices increased from the early to mid-second trimester, followed by a slight fall in the late second trimester. Notably, higher iron indices were observed in mothers of preterm and low-birth-weight neonates.

## 1. Introduction

Iron deficiency anemia (IDA) remains a significant public health concern, particularly in low- and middle-income countries. Pregnant women are at increased risk of IDA due to specific pathophysiological mechanisms [1]. Those affected by IDA during pregnancy are more susceptible to complications, such as perinatal infections, pre-eclampsia, cardiac failure, and hemorrhagic events, which, in severe cases, may lead to maternal mortality [2,3]. Moreover, IDA is associated with high rates of perinatal morbidity and mortality [4,5]. A recent meta-analysis reported that maternal anemia is linked to an 18% increase in perinatal mortality and a 20% rise in maternal mortality in South Asian countries, including India [6].

Iron plays a crucial role in fetal development, facilitating rapid cellular proliferation and brain myelination [7,8]. Maternal iron deficiency is associated with adverse neonatal outcomes, including preterm birth, low birth weight, and intrauterine growth restriction, as well as increased risks of cognitive delays [9], autism, learning disabilities, neurodevelopmental disorders [10], and metabolic syndrome in adulthood [2]. Newborns with low iron stores require immediate evaluation and intervention, as untreated iron deficiency can have long-term consequences, diminished cognitive function, and impaired immune system development [11,12]. However, the impact of maternal IDA on fetal iron status remains controversial. Previous evidence reported by Rios et al. [7] and Van Eijk et al. [8], suggests that the fetus absorbs iron from the mother regardless of her iron levels [7,8], while others reported that maternal iron deficiency leads to lower fetal and neonatal iron stores [13,14]. As maternal iron serves as the primary source of iron for the fetus, assessing the prevalence of IDA and its consequences is crucial [15]. Research indicates that term infants born to mothers with IDA often have inadequate iron stores, increasing their risk of anemia [15,16].

Additionally, evidence consistently highlights a strong correlation between maternal hemoglobin levels and adverse birth outcomes [17]. A recent systematic review found that iron deficiency during the first and second trimesters significantly heightens the risk of unfavorable pregnancy outcomes and maternal morbidity [18]. Several studies have investigated the impact of maternal hematological status, particularly hemoglobin and serum ferritin levels, on the iron reserves of newborns and pregnancy outcomes; however, the findings have been inconsistent. Therefore, the main objective of this study was to correlate maternal iron indices in the second trimester with cord blood indices and pregnancy outcomes. We hypothesized that lower maternal iron indices are significantly linked to decreased cord iron indices and an increased risk of adverse outcomes. Understanding this relationship is essential for early interventions and improving maternal and neonatal health.

## 2. Materials and Methods

### 2.1. Study Design

The present study was a prospective cohort study nested within the RAPIDIRON Trial [19] at the Jawaharlal Nehru Medical College Women’s and Children’s Health Research unit, Karnataka, India. Pregnant women who had moderate anemia and subsequently received oral iron supplementation were eligible for enrolment. This study (KAHER/EC/21-22/001) was approved on 29th March 2021 by the Institutional Ethics Committee of the KLE Academy of Higher Education and Research (KAHER), Belagavi.

### 2.2. Participants

We included consenting pregnant women between the ages of 18 and 40 years from the rural CHC and PHC in and around Belgaum in the early second trimester. An Hb concentration of 7.0–9.9 g/dL defined moderate anemia and represented an inclusion criterion. Maternal participants with a twin pregnancy or any congenital anomaly diagnosed at dating ultrasound were excluded.

### 2.3. Sample Size

The sample size was estimated assuming a prevalence of 37.3% of anemia in pregnant women [20], with a confidence interval of 95% corresponding to 1.96 alpha, and a 15% margin of error, resulting in a calculated sample size of 287.

### 2.4. Study Procedure

All pregnant women were screened initially in the first trimester, and blood samples were collected in the early second trimester at 12–16 weeks of gestation. Pregnant women with hemoglobin levels ranging from 7.0 to 9.9 g/dL, serum ferritin < 30 ng/mL, and/or TSAT < 20% were eligible for this study. As a part of the RAPIDIRON Trial, participants were recommended to take 60 mg of ferrous sulfate twice a day and 400 mcg of folic acid once a day throughout pregnancy. A pregnant participant who consented to this study was contacted in the mid- (20–24 weeks) and late (26–30 weeks) second trimester, and a maternal blood sample as well as a cord blood sample were collected following delivery. Also, a detailed history of maternal and neonatal outcomes was noted. The term “Pregnancy outcomes” encompasses instances where current pregnancies result in a low birth weight, preterm birth, or stillbirth. All anthropometric measurements (including birth weight and birth length) and dietary habit data were retrieved from the RAPIDIRON Trial dataset [19].

### 2.5. Laboratory Investigation

A 2 mL sample of both maternal and cord blood was collected in EDTA and plain vacutainers for whole-blood and serum analysis in respective visits. Serum aliquots were separated and stored at −80 °C for sTfR assay until analysis. sTfR concentrations were measured by the sandwich ELISA method. The serum analysis, including ferritin and transferrin saturation (TSAT), was immediately analyzed by Roche-Cobas-6000 [ECLIA]. The whole-blood analysis, including hemoglobin, reticulocyte hemoglobin (Ret-Hb), immature reticulocyte fraction (IRF), mean corpuscular volume (MCV), mean corpuscular hemoglobin (MCH), and mean corpuscular hemoglobin concentration (MCHC), was analyzed with a Sysmex-Hematology analyzer.

### 2.6. Statistical Analysis

All the categorical variables were summarized using frequencies and percentages. All the continuous variables were summarized using mean (SD)/median (Q1; Q3) values depending on the normality of the data. The normality assumption was assessed using the Kolmogorov–Smirnov test. Karl Pearson’s/Spearman’s rank correlation coefficient was used to find the association between maternal iron indices and cord blood iron indices. Mixed ANOVA (analysis of variance) was used to compare the trend in maternal Hb, TSAT, ferritin, and sTfR levels over time across different groups. Mauchly’s test was employed to assess the sphericity of the data, and Greenhouse–Geisser-corrected significance values were used when sphericity was lacking. Post hoc analysis was performed with Bonferroni’s correction for multiple comparisons. A comparison of trends in maternal iron indices across the two dietary groups was performed using the Mann–Whitney U test. All the statistical analysis was carried out using SPSS version 16. A *p*-value of less than 0.05 was considered indicative of statistical significance for all analyses. Outcomes were categorized as follows: neonatal anemia was anemic (Hb < 13.0 g/dL) or non-anemic (Hb ≥ 13.0 g/dL), birth weight was low birth weight (<2.5 Kgs) or normal birth weight (≥2.5 Kgs), gestational age at the time of birth was preterm birth (<37 weeks) or term birth (≥37 weeks), and the diet type was vegetarian or a mixed diet.

## 3. Results

Of the 315 participants enrolled, a cohort of 292 mothers was included in the final analysis of this study, as 23 cord blood samples could not be collected and were lost to follow-up. A subsample of 105 subjects was analyzed to determine soluble transferrin receptor (sTfR) levels at each time point. Key maternal and newborn characteristics, complications, and cord blood variables are outlined in Table 1.

**Figure 1:**
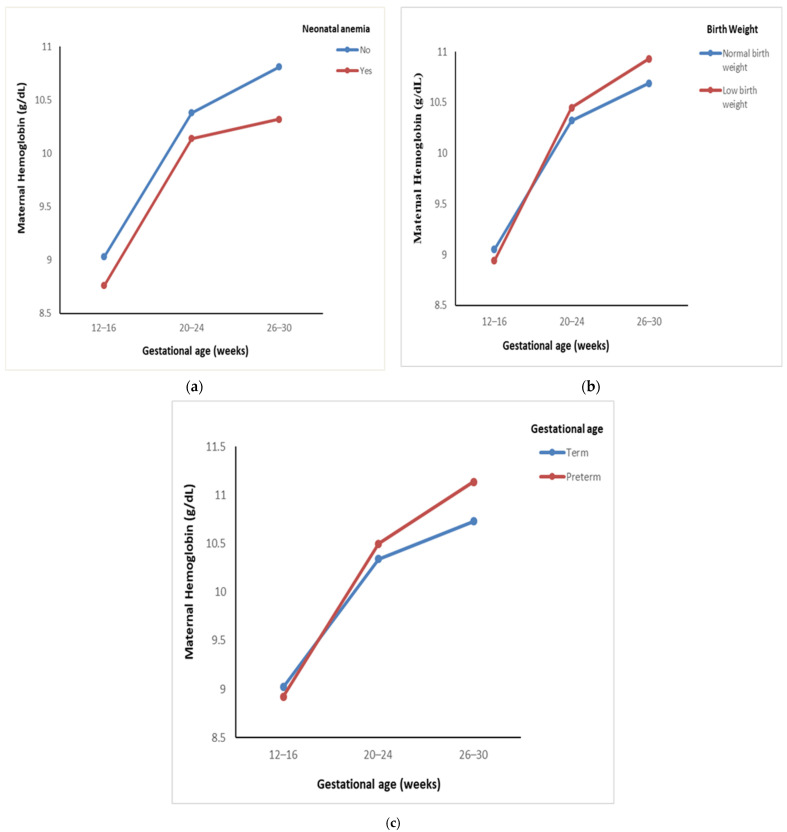
Comparison of trends in maternal hemoglobin at different gestational ages across pregnancy outcomes: (**a**) trends in maternal hemoglobin with neonatal anemia; (**b**) trends in maternal hemoglobin across birth weights; (**c**) trends in maternal hemoglobin across gestational ages at birth.

The comparison of trends in maternal hemoglobin levels across gestational ages and pregnancy outcomes is represented in Table 2. A significant increase in maternal hemoglobin levels was observed in mothers of both anemic and non-anemic neonates. In mothers of anemic neonates, hemoglobin levels increased from 8.76 to 10.32 g/dL, while in mothers of non-anemic neonates, levels rose from 9.03 to 10.81 g/dL (*p* < 0.001). Despite the overall increase, the rise in hemoglobin levels was gradual in mothers of anemic neonates, with no significant difference between groups (Figure 1a). Additionally, mothers of low-birth-weight neonates exhibited a significant increase in hemoglobin levels from 8.94 to 10.93 g/dL, compared with an increase from 9.05 to 10.69 g/dL in mothers of normal-weight neonates (*p* < 0.001). An interaction F-value of 3.28 (*p* = 0.05) was also observed (Figure 1b). A significant increase in hemoglobin levels was also observed in mothers of both preterm and term neonates (*p* < 0.001), with a trend toward higher levels in mothers of preterm neonates (Figure 1c).

Post hoc comparisons adjusted using Bonferroni corrections were performed on maternal iron indices (hemoglobin, TSAT, ferritin, and sTfR) across the gestational periods (12–16, 20–24, and 26–30 weeks) and pregnancy outcome groups. A significant difference in hemoglobin levels was observed between the pregnancy outcome groups (*p* < 0.001). However, for mothers of anemic neonates, there was no significant difference in hemoglobin levels between 20–24 and 26–30 weeks (*p* = 0.69).

**Figure 2:**
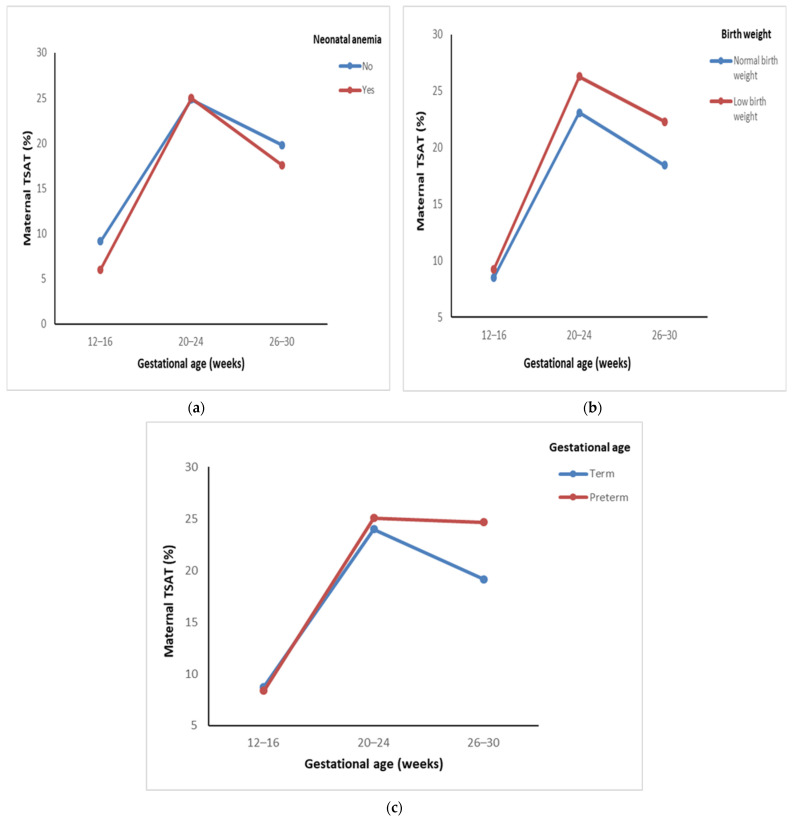
Comparison of trends in maternal TSAT at different gestational ages across pregnancy outcomes: (**a**) trends in maternal TSAT with neonatal anemia; (**b**) trends in maternal TSAT across birth weights; (**c**) trends in maternal TSAT across gestational ages at birth.

The comparison of trends in maternal transferrin saturation (TSAT) levels across gestational ages and pregnancy outcomes is represented in Table 3. A significant increase in maternal TSAT levels was observed from baseline to the mid-second trimester, followed by a slight decline in the late second trimester across all groups, with all changes being statistically significant (*p* < 0.001). In mothers of anemic neonates, TSAT levels rose significantly from 6.02% to 17.57%, while in mothers of non-anemic neonates, levels increased from 9.14% to 19.79% (Figure 2a). For mothers delivering a low-birth-weight neonate, TSAT levels rose from 9.24% to 22.30%, higher compared with an increase from 8.52% to 18.44% in mothers delivering normal-weight neonates (Figure 2b). Similarly, TSAT levels were higher in mothers with preterm deliveries, where they increased from 8.36% to 24.67% compared with 8.78% to 19.14% in those with term deliveries (Figure 2c). No significant differences were observed between the groups in pregnancy outcomes.

Post hoc analysis for TSAT levels among mothers of non-anemic, normal-weight, term-birth neonates showed a significant difference at each gestational week (*p* < 0.001). The TSAT levels in mothers of anemic, low-birth-weight, preterm neonates showed no significant difference across 20–24 to 26–30 weeks of gestation (*p* = 0.19, 0.16, and 1.00).

The comparison of trends in maternal ferritin levels across gestational ages and pregnancy outcomes is represented in Table 4. A pattern similar to maternal TSAT was observed, with ferritin levels significantly increasing from baseline to the mid-second trimester, followed by a slight decline in the late second trimester across all groups. All changes in ferritin levels were statistically significant (*p* < 0.001). In mothers of non-anemic neonates, ferritin levels increased throughout the second trimester from 11.15 to 35.44 ng/mL, whereas mothers of anemic neonates exhibited comparatively lower ferritin levels, increasing from 8.86 to 30.51 ng/mL.

Mothers of low-birth-weight neonates showed an increase from 12.93 to 36.52 ng/mL, while in mothers of normal-birth-weight neonates, levels rose from 11.62 to 33.51 ng/mL. Notably, ferritin levels were higher in mothers of low-birth-weight neonates compared with those delivering normal-birth-weight neonates. Similarly, ferritin levels were higher in mothers of preterm neonates, increasing from 15.64 to 40.71 ng/mL, compared with 11.67 to 33.83 ng/mL in mothers of term neonates. No significant differences were observed between pregnancy outcome groups (Supplementary Figure S1).

The post hoc analysis for ferritin levels revealed that there was a significant difference observed between mothers of all pregnancy outcome groups across all weeks of gestation (*p* < 0.001). However, no significant difference was observed between 20–24 and 26–30 weeks (*p* = 1.00).

The trends in maternal soluble transferrin receptor (sTfR) levels across different gestational ages and pregnancy outcomes are depicted in Table 5. The findings indicate a statistically significant improvement in sTfR levels across all groups (*p* < 0.001). In mothers with anemic neonates, levels declined from 7.72 to 5.87 µg/mL, while those with non-anemic neonates showed a reduction from 7.51 to 5.76 µg/mL. Similar trends were observed in mothers of low-birth-weight (7.41 to 5.79 µg/mL) and normal-birth-weight neonates (7.62 to 5.79 µg/mL). Additionally, sTfR levels decreased in mothers of preterm (7.74 to 6.04 µg/mL) and term neonates (7.54 to 5.78 µg/mL). The graphical representation of the trend in maternal sTfR levels across gestational ages and outcomes is provided (Supplementary Figure S2).

The post hoc analysis for sTfR levels showed significant differences between mothers in all pregnancy outcome groups across all weeks of gestation (*p* < 0.001). The maternal iron indices showed a negligible correlation with the cord blood iron indices. The results are provided in Supplementary Table S1.

A longitudinal comparison of maternal iron indices (Hb, TSAT, ferritin, and sTfR) across three gestational time points (12–16, 20–24, and 26–30 weeks) stratified by maternal diet type (vegetarian vs. mixed diet) was performed. Both dietary groups showed significant improvements in all iron indices over time (*p* < 0.001), indicating a positive response to oral iron supplementation. However, no significant interaction was observed between diet type and gestational age for any of the indices, suggesting that maternal dietary habits did not influence the trajectory of iron status during the second trimester. Hemoglobin levels increased steadily in both groups, TSAT and ferritin increased from the early to mid-trimesters before stabilizing, and sTfR levels declined significantly, reflecting improved iron availability (*p* < 0.001). These findings highlight that the efficacy of iron supplementation was comparable across both dietary groups, supporting its utility in improving maternal iron status regardless of dietary preference. The results are provided in Supplementary Table S2 and Figure S3.

Supplementary Table S3 compares cord blood iron parameters between neonates born to vegetarian and mixed-diet mothers. The results show no statistically significant differences in any of the measured indices between the two dietary groups. The median cord blood Hb levels were similar in neonates of vegetarian and mixed-diet mothers (14.99 g/dL vs. 15.18 g/dL; *p* = 0.39), alongside TSAT levels (57.02% vs. 59.03%; *p* = 0.19), ferritin levels (233 ng/mL vs. 211.90 ng/mL; *p* = 0.40), and sTfR levels (8.15 µg/mL vs. 7.89 µg/mL, *p* = 0.47). These findings indicate that maternal diet type did not significantly impact neonatal iron status at birth.

## 4. Discussion

### 4.1. The Main Findings

This study observed a substantial increase in maternal hemoglobin, TSAT, ferritin, and sTfR levels throughout pregnancy. Across all three visits, maternal hemoglobin levels were higher in non-anemic neonates than in anemic neonates, suggesting that elevated prenatal hemoglobin corresponds to higher neonatal hemoglobin concentrations. Notably, term and normal-birth-weight neonates exhibited lower maternal hemoglobin at the second and third visits, suggesting that adverse delivery outcomes may occur despite adequate maternal hemoglobin levels. Furthermore, maternal ferritin and TSAT followed similar trends, while a significant decrease in sTfR levels indicated improved iron status and normal erythropoietic activity.

A study reported that neonates born to anemic mothers had considerably lower Hb values (*p* < 0.05) than those born to non-anemic mothers [21]. Our investigation found a similar relationship between lower maternal Hb values and lower Hb in neonates. In contrast, a study found no relationship between maternal and newborn Hb levels (Pearson correlation: −0.01) [22]. Several studies found no link between maternal Hb levels, neonatal iron status, and risk of preterm birth. Despite low Hb levels in the first trimester and an increase in the second, maternal Hb was not associated with a higher risk of preterm delivery [23,24], suggesting that Hb levels may not directly influence adverse birth outcomes.

Preterm births may occur in mothers with normal Hb levels due to other factors, such as thyroid dysfunction, placenta previa, or a history of preterm births [25]. Similar findings were observed in our study, where preterm neonates had higher maternal Hb levels than term newborns. Elevated Hb levels in the second and third trimesters were associated with an increased risk of preterm birth and small-for-gestational-age (SGA) infants, while high Hb levels in the first and second trimesters were associated with SGA but not preterm birth [26]. This correlates with the elevated maternal Hb levels in our study’s preterm and LBW newborns. In contrast with our study, Smith et al. reported that a reduced risk of spontaneous preterm birth was associated with low Hb levels in the third trimester [27].

A high Hb concentration in the early third trimester may indicate insufficient plasma volume expansion, increasing blood viscosity and impairing placental perfusion [28,29,30]. This reduces fetal oxygen and nutrient delivery, compromising growth and development [31,32]. As a result, elevated hemoglobin levels during pregnancy are linked to a higher risk of adverse outcomes. Thus, proper plasma volume expansion is crucial for promoting positive pregnancy outcomes. An inverse relationship was found between birth weight and higher maternal ferritin levels during the second trimester [33]. Another study reported that there was a significant correlation between low birth weight and preterm birth when there was a high serum ferritin level in the third trimester [34]. Similarly, a Chinese study identified a correlation between elevated ferritin levels in the second trimester and an increased incidence of preterm birth and low birth weight [35,36]. Similar findings were also observed in our study, which showed that preterm and low-birth-weight neonates had higher maternal ferritin concentrations than term and normal-birth-weight neonates.

Elevated ferritin levels in the second or third trimester have been linked to adverse pregnancy outcomes [37,38], possibly indicating inflammation or inadequate plasma volume expansion. Excess iron surpasses transferrin capacity, leading to non-transferrin-bound iron, which induces oxidative stress, lipid peroxidation, and DNA damage in placental cells. This disrupts immune responses and fetal growth, contributing to adverse maternal and neonatal outcomes [39,40]. A comparative study investigating the iron status of pregnant and non-pregnant women found a significant increase in iron and TSAT levels from the first to the third trimester [41]. Similarly, in our study, we observed a significant increase in maternal TSAT levels from the early to mid-second trimester, followed by a slight decline in the late second trimester across all outcome groups.

Serum sTfR levels are raised when membrane transferrin receptors are activated to improve iron uptake into cells when tissue iron availability is low [42]. A study on iron supplementation in women with iron deficiency without anemia found significant reductions in sTfR levels and a rise in serum ferritin and hemoglobin levels [43]. Similarly, another study reported improved sTfR and TIBC levels [44]. In our study, sTfR levels significantly improved from the early to late second trimester of pregnancy. We found only a negligible correlation between maternal and cord blood iron indices. Similarly, findings were also observed in another study [21]. However, in contrast with our findings, another study demonstrated a significant relationship between maternal and cord blood iron, ferritin, sTfR, and the sTfR/log ferritin index [45,46].

A study on the effect of dietary habits on anemia prevalence in pregnant women found no significant association between anemia and being vegetarian or consuming meat [47]. Similarly, another study assessing maternal diets and iron status reported no significant differences in maternal or umbilical cord levels of B12, folate, ferritin, or hemoglobin between groups [48]. Although our study observed significant improvements in maternal iron indices over time in both dietary groups, we did not find any significant interaction between dietary groups and maternal or cord blood iron indices, indicating that maternal diet did not influence the trajectory of iron status during the second trimester.

### 4.2. Clinical Implications

This study underscores the importance of assessing and managing maternal iron status during the second trimester to improve neonatal iron stores and pregnancy outcomes. The findings support the integration of routine screening of maternal iron indices into antenatal care protocols, particularly in rural and resource-limited settings. Such measures would enable the early identification of pregnant women at risk for iron deficiency and allow for timely and targeted interventions. These strategies have the potential to enhance the overall quality of maternal health, improve fetal iron status, and reduce the incidence of adverse outcomes, such as low birth weight and neonatal iron deficiency.

### 4.3. Strengths, Limitations, and Future Directions

A key strength and limitation of this study was its exclusive focus on pregnant women with moderate anemia receiving oral iron supplementation, providing valuable insights into maternal and neonatal outcomes. Unlike most studies assessing iron parameters (Hb/ferritin) at limited time points, we evaluated iron indices longitudinally across pregnancy, incorporating maternal and cord blood variables. The inclusion of sTfR enhances our understanding of erythropoiesis and iron status. Our comprehensive analysis of maternal, neonatal, and obstetric characteristics and dietary habits distinguishes this research. However, we did not assess socio-economic, environmental, or genetic factors or other micronutrients. Also, hepcidin levels, which are a key regulator of placental iron transport and maternal–fetal iron transfer, were not analyzed, as we focused on sTfR, which was measurable, and it is a reliable marker of cellular iron demand and is less influenced by inflammation, making it especially suitable in populations with a high infectious burden. Future research should focus on longitudinal, multicentric studies that incorporate markers of inflammation and genetic factors influencing iron metabolism. Investigating the long-term neurodevelopmental outcomes of neonates born to iron-deficient mothers may also provide a deeper understanding of the implications of prenatal iron status.

## 5. Conclusions

This study observed a positive trend in all the maternal iron indices in the early to mid-second trimester and a slight decline in the late second trimester among each outcome group. Surprisingly, we found higher levels of iron indices in mothers of preterm and low-birth-weight neonates than in mothers of term and normal-birth-weight neonates. Our findings emphasize the importance of monitoring and managing iron levels in pregnant women throughout pregnancy in the hope of improving maternal and neonatal health.

## Figures and Tables

**Table 1:** Study population characteristics (*n* = 292).

Maternal and Newborn Characteristics		Mean ± SD/Median (Q1, Q3)/*n* (%)
**Maternal characteristics**		
Maternal age (years)		22 (20, 26)
Maternal Hb (at delivery) (g/dL)		12.21 (11.40, 12.72)
Gestational age (at delivery)	Term birth	268 (91.78)
	Preterm birth	24 (8.22)
Mode of delivery	Vaginal delivery	172 (58.90)
	C-section	120 (41.10)
**Newborn characteristics**		
Gender	Male	149 (51.03)
	Female	143 (48.97)
Birth weight	Normal birth weight	202 (69.18)
	Low birth weight	90 (30.82)
**Cord blood RBC indices**		
MCV (fL)		107.63 ± 6.42
MCH (pg/cell)		34.50 (33.40, 35.60)
MCHC (g/dL)		31.90 (31.40, 32.50)
IRF (%)		29.10 (23.90, 33.30)
Ret-Hb (pg)		32.30 (31.10, 33.30)
**Pregnancy complications**		
Prolonged labor		48 (16.44)
Antepartum hemorrhage		6 (2.05)
Severe postpartum hemorrhage		1 (0.34)
Eclampsia		16 (5.48)
Maternal anemia		47 (16.2)
Stillbirths		4 (1.37)
Newborns admitted to NICU		15 (5.14)

**Table 2:** Comparison of trends in maternal hemoglobin (mean ± SD) at different gestational ages across pregnancy outcomes.

Pregnancy Outcome			Maternal Hb (g/dL)			Interaction F-Value (*p*-Value)	Within-Group F-Statistic	*p*-Value
		*n*	12–16 GA	20–24 GA	26–30 GA			
Neonatal anemia	Yes	27	8.76 ± 0.79	10.14 ± 1.03	10.32 ± 1.13	0.62 ^$^	19.59	<0.001 *
	No	253	9.03 ± 0.78	10.38 ± 1.02	10.81 ± 1.26	(0.49)	203.46	<0.001 *
Low birth weight	Yes	97	8.94 ± 0.75	10.45 ± 1.11	10.93 ± 1.39	3.28 ^$^	100.77	<0.001 *
	No	218	9.05 ± 0.78	10.32 ± 0.99	10.69 ± 1.18	(0.05)	155.71	<0.001 *
Preterm birth	Yes	28	8.92 ± 0.81	10.50 ± 1.07	11.14 ± 1.31	2.52 ^$^	35.00	<0.001 *
	No	287	9.02 ± 0.77	10.34 ± 1.03	10.73 ± 1.24	(0.10)	220.26	<0.001 *

^$^ Adjusted for Greenhouse–Geisser correction; * statistically significant.

**Table 3:** Comparison of trends in maternal TSAT (mean ± SD) at different gestational ages across pregnancy outcomes.

Pregnancy Outcome			Maternal TSAT (%)			Interaction F-Value (*p*-Value)	Within-Group F-Statistic	*p*-Value
		*n*	12–16 GA	20–24 GA	26–30 GA			
Neonatal anemia	Yes	27	6.02 ± 2.66	24.99 ± 16.96	17.57 ± 11.92	0.37 ^$^	12.77	<0.001 *
	No	253	9.14 ± 10.14	24.83 ± 19.48	19.79 ± 15.14	(0.68)	87.71	<0.001 *
Low birth weight	Yes	97	9.24 ± 12.26	26.27 ± 25.13	22.30 ± 17.94	1.03 ^$^	47.48	<0.001 *
	No	218	8.52 ± 7.69	23.09 ± 14.75	18.44 ± 13.02	(0.36)	70.90	<0.001 *
Preterm birth	Yes	28	8.36 ± 7.80	25.07 ± 15.75	24.67 ± 19.96	1.37 ^$^	17.21	<0.001 *
	No	287	8.78 ± 9.47	23.97 ± 18.86	19.14 ± 14.14	(0.26)	101.32	<0.001 *

^$^ Adjusted for Greenhouse–Geisser correction; * statistically significant.

**Table 4:** Comparison of trends in maternal ferritin (mean ± SD) at different gestational ages across pregnancy outcomes.

Pregnancy Outcome			Maternal Ferritin (ng/mL)			Interaction F-Value (*p*-Value)	Within-Group F-Statistic	*p*-Value
		*n*	12–16 GA	20–24 GA	26–30 GA			
Neonatal anemia	Yes	27	8.86 ± 8.49	31.52 ± 16.47	30.51 ± 28.54	0.10	6.68	0.001 *
	No	253	11.15 ± 15.06	36.87 ± 38.72	35.44 ± 34.28	(0.90)	79.63	<0.001 *
Low birth weight	Yes	97	12.93 ± 18.77	39.86 ± 49.53	36.52 ± 29.82	0.32	31.00	<0.001 *
	No	218	11.62 ± 21.09	35.03 ± 30.23	33.51 ± 33.70	(0.72)	55.70	<0.001 *
Preterm birth	Yes	28	15.64 ± 21.50	51.67 ± 83.26	40.71 ± 37.44	1.71	14.08	<0.001 *
	No	287	11.67 ± 20.28	35.04 ± 29.03	33.83 ± 32.03	(0.18)	74.30	<0.001 *

* statistically significant.

**Table 5:** Comparison of trends in maternal sTfR (mean ± SD) at different gestational ages across pregnancy outcomes.

Pregnancy Outcome			Maternal sTfR (µg/mL)		Interaction F-Value (*p*-Value)	Within-Group F-Statistic	*p*-Value
		*n*	12–16 GA	26–30 GA			
Neonatal anemia	Yes	13	7.72 ± 1.33	5.87 ± 0.81	0.04 ^$^	14.68	0.001 *
	No	91	7.51 ± 1.61	5.76 ± 1.11	(0.85)	91.89	<0.001 *
Low birth weight	Yes	34	7.41 ± 1.50	5.79 ± 1.18	0.33 ^$^	29.81	<0.001 *
	No	71	7.62 ± 1.62	5.79 ± 1.04	(0.57)	79.20	<0.001 *
Preterm birth	Yes	5	7.74 ± 1.13	6.04 ± 1.06	0.01 ^$^	4.82	0.03 *
	No	100	7.54 ± 1.60	5.78 ± 1.09	(0.94)	103.52	<0.001 *

^$^ Adjusted for Greenhouse–Geisser correction; * statistically significant.

## Data Availability

The original contributions presented in this study are included in this article/the Supplementary Material. Further inquiries can be directed to the corresponding author.

## Supplementary Materials

The following supporting information can be downloaded at https://www.mdpi.com/article/10.3390/nu17091584/s1. Figure S1: Comparison of trends in maternal ferritin at different gestational ages across pregnancy outcomes. Figure S2: Comparison of trends in maternal sTfR at different gestational ages across pregnancy outcomes. Table S1: Correlation between maternal iron indices at each gestational age in the second trimester with cord blood iron indices. Figure S3: Comparison of trends in maternal iron indices: (a) Hb, (b) TSAT, (c) ferritin, and (d) sTfR across gestational ages by maternal diet type (vegetarian vs. mixed). Table S2: Comparison of trends in maternal iron indices (mean ± SD) across gestational ages by maternal diet type (vegetarian vs. mixed). Table S3: Comparison of cord blood iron parameters by maternal diet type (vegetarian vs. mixed).
