# Supplementary material for: Relationship Between Maternal Iron Indices in the Second Trimester with Cord Blood Iron Indices and Pregnancy Outcomes: A Prospective Cohort Study
Source: Nutrients. 2025 May 5;17(9):1584. doi: 10.3390/nu17091584 (PMC12073715; doi:10.3390/nu17091584)
Supplement: Supplementary file 1 [file nutrients-17-01584-s001.zip › Supplementary_Figure_S1.pdf]

**Supplementary Figure S1.** Comparison of trends in maternal ferritin over different gestational ages across pregnancy outcomes: (a) Trends in maternal ferritin across neonatal anemia; (b) Trends in maternal ferritin across birth weight; (c) Trends in maternal ferritin across gestational age at birth.

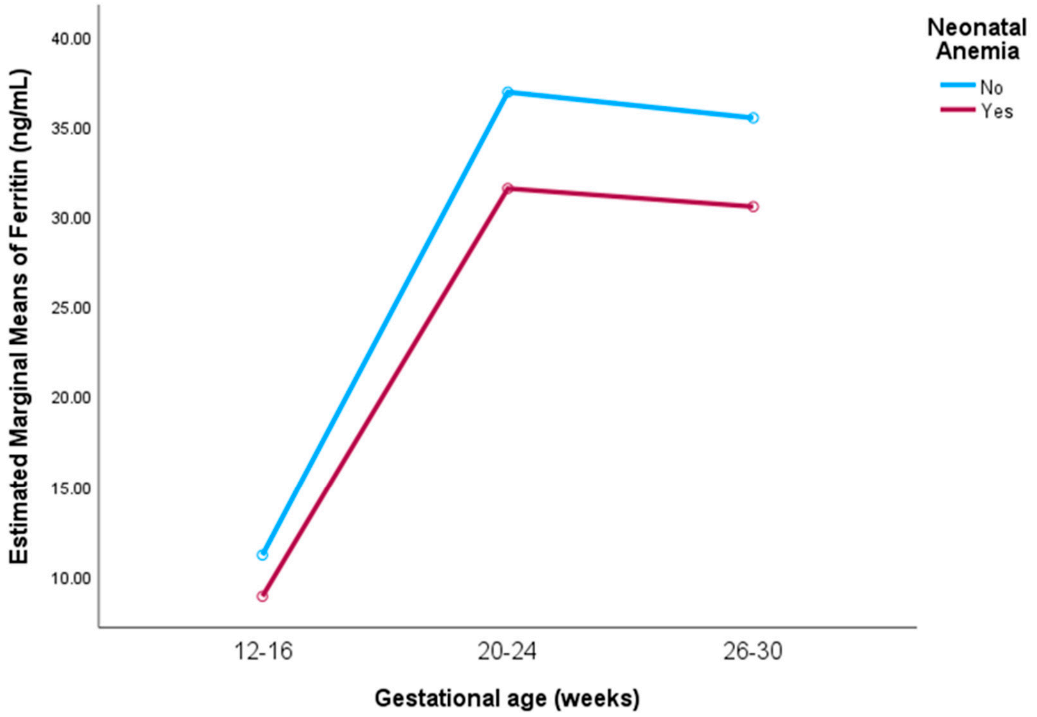


(a)

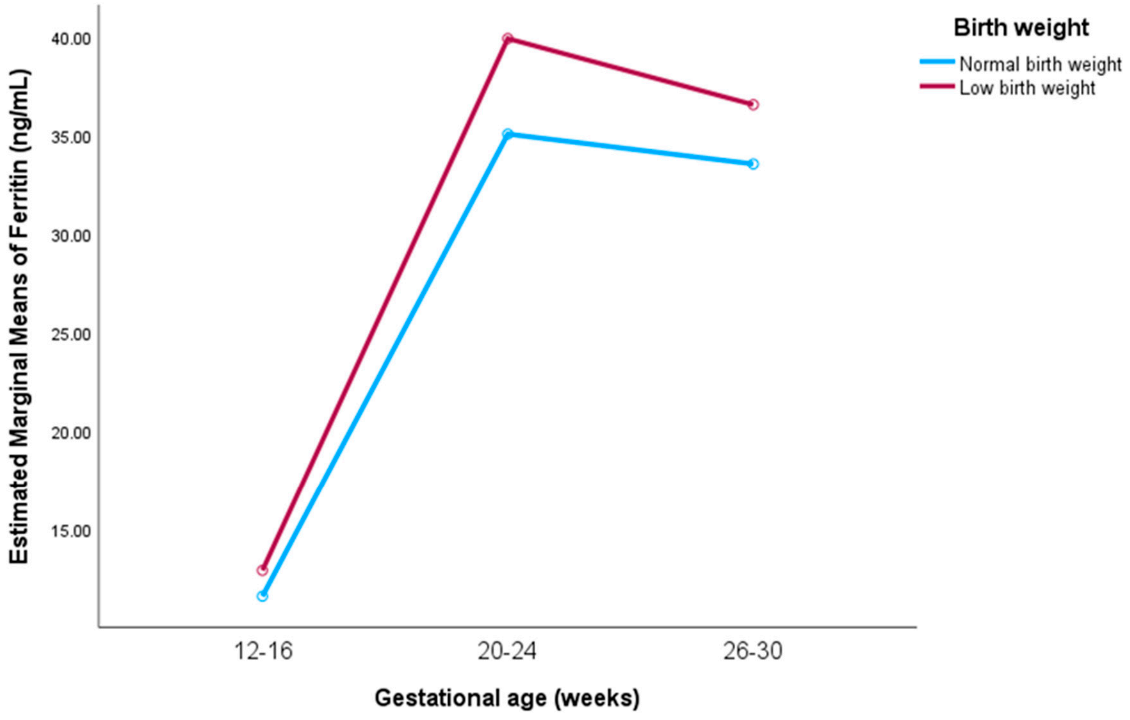


(b)

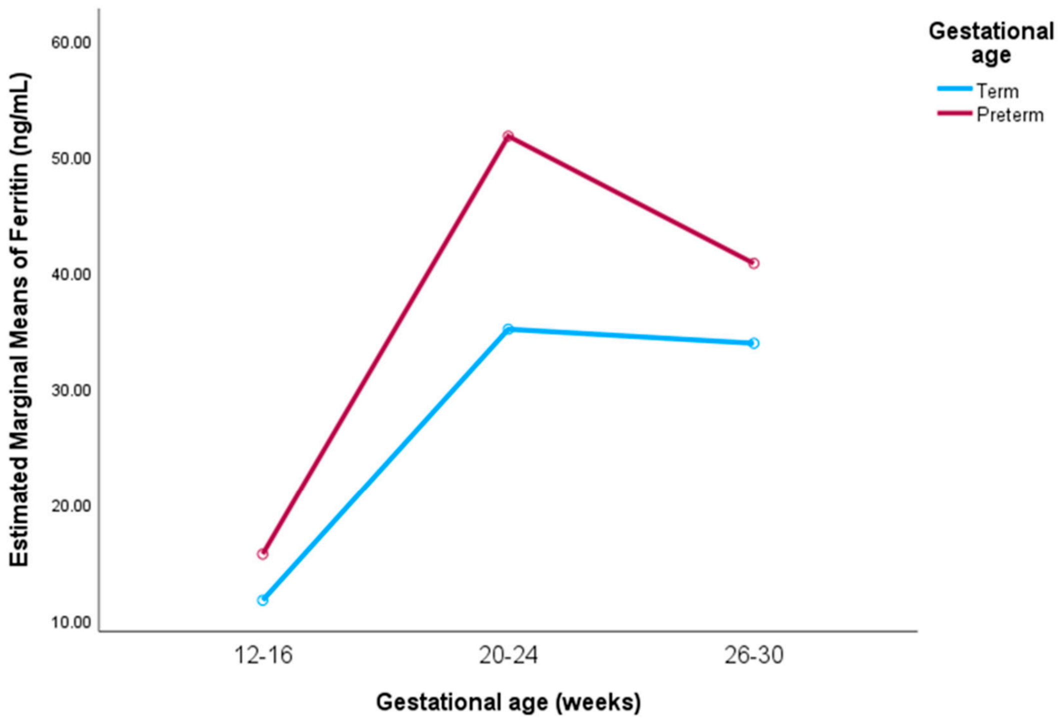

Estimated Marginal Means of Ferritin (ng/mL)
60.00
50.00
40.00
30.00
20.00
10.00
12-16
20-24
26-30
Gestational age (weeks)
Gestational age
Term
Preterm


(c)
