# Supplementary material for: Relationship Between Maternal Iron Indices in the Second Trimester with Cord Blood Iron Indices and Pregnancy Outcomes: A Prospective Cohort Study
Source: Nutrients. 2025 May 5;17(9):1584. doi: 10.3390/nu17091584 (PMC12073715; doi:10.3390/nu17091584)
Supplement: Supplementary file 1 [file nutrients-17-01584-s001.zip › Supplementary_Figure_S2.pdf]

Supplementary Figure S2. Comparison of trends in maternal sTfR over different gestational ages across pregnancy outcomes: (a) Trends in maternal sTfR across neonatal anemia; (b) Trends in maternal sTfR across birth weight; (c) Trends in maternal sTfR across gestational age at birth.

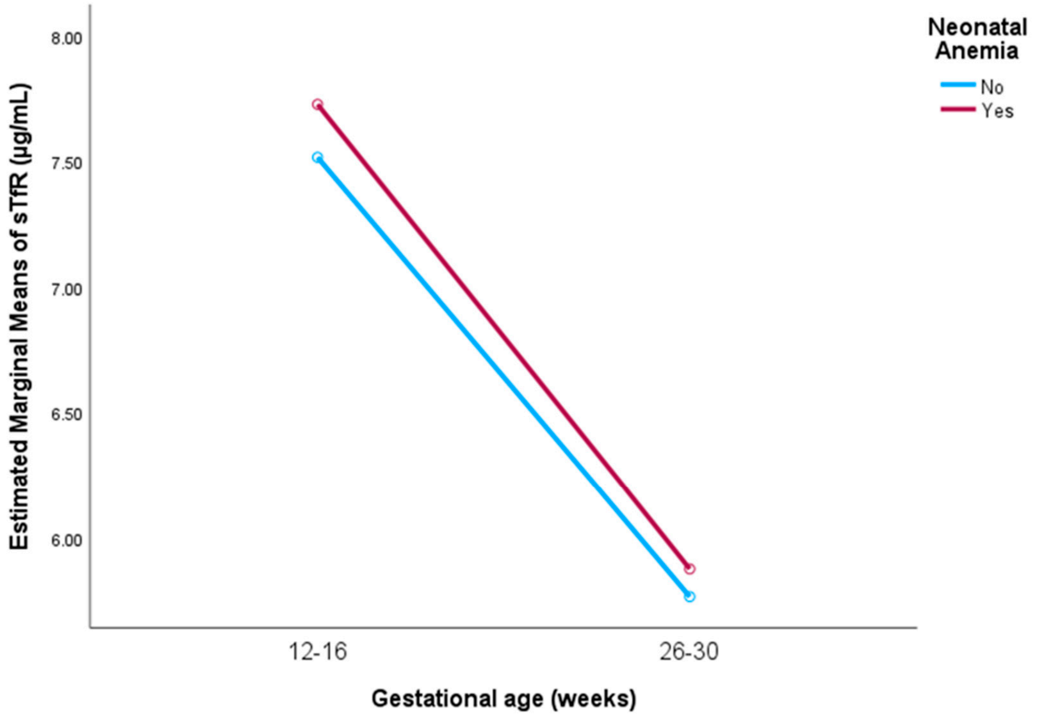

(a)

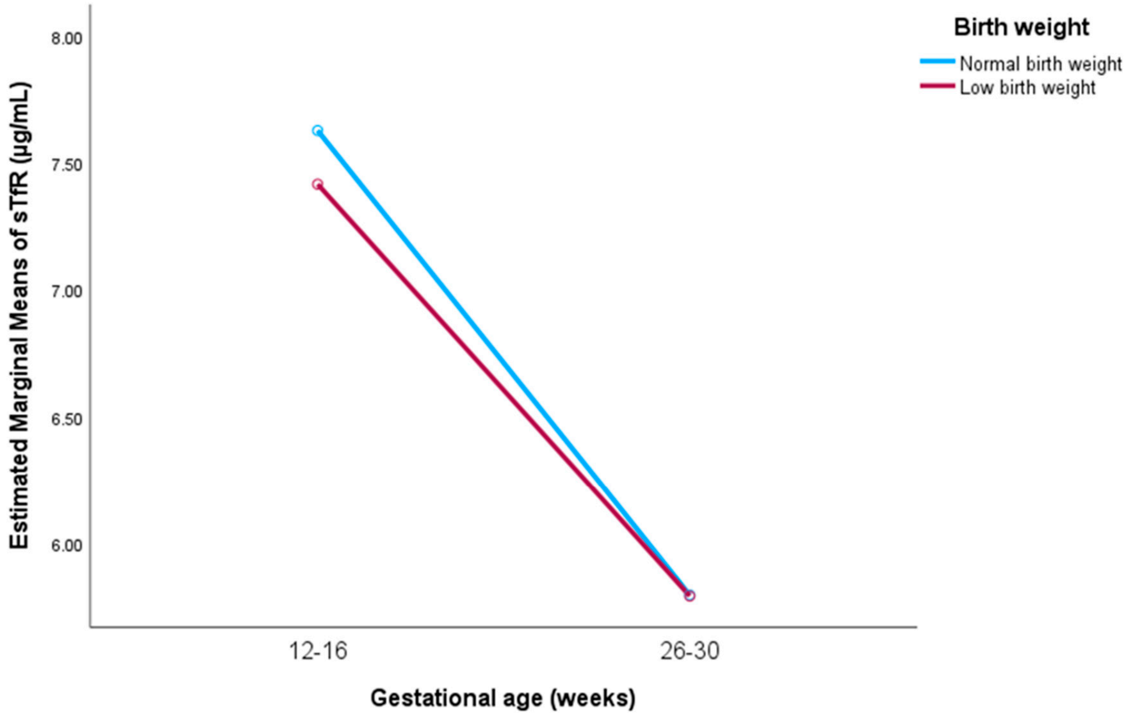

(b)

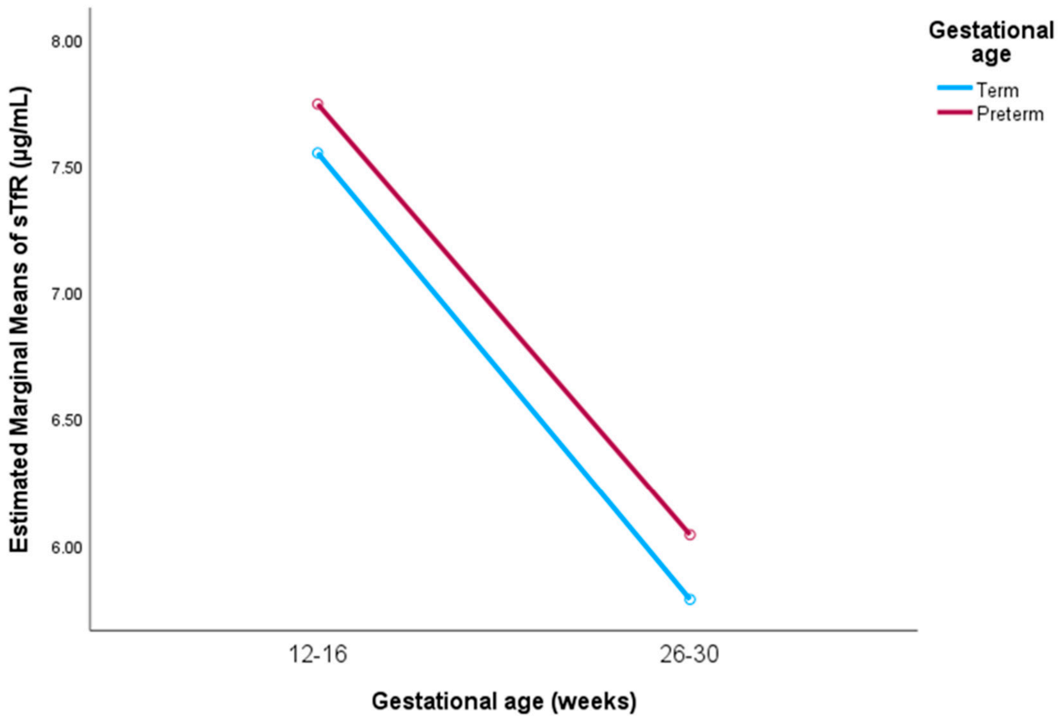
Estimated Marginal Means of sTfR (µg/mL)
8.00
7.50
7.00
6.50
6.00
12-16
26-30
Gestational age (weeks)
Gestational age
Term
Preterm

(c)
