# Supplementary material for: Relationship Between Maternal Iron Indices in the Second Trimester with Cord Blood Iron Indices and Pregnancy Outcomes: A Prospective Cohort Study
Source: Nutrients. 2025 May 5;17(9):1584. doi: 10.3390/nu17091584 (PMC12073715; doi:10.3390/nu17091584)
Supplement: Supplementary file 1 [file nutrients-17-01584-s001.zip › Supplementary_Figure_S3.pdf]

Supplementary Figure S3. Comparison of trends in maternal iron indices (a) Hb, (b) TSAT, (c) Ferritin, (d) sTfR across gestational age by maternal diet type (vegetarian vs. mixed).

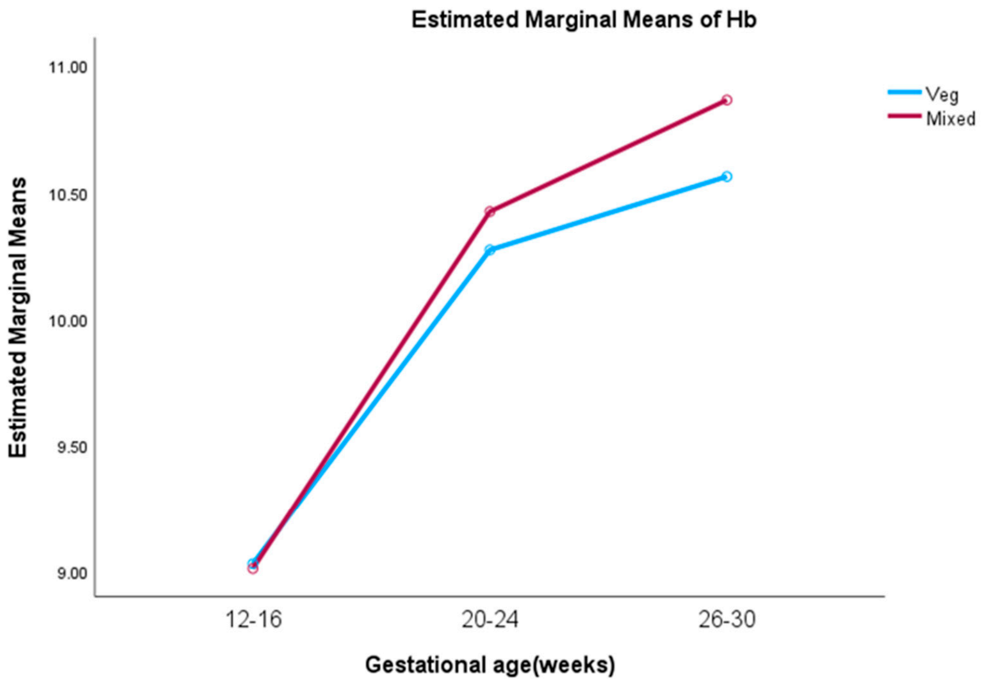

(a)

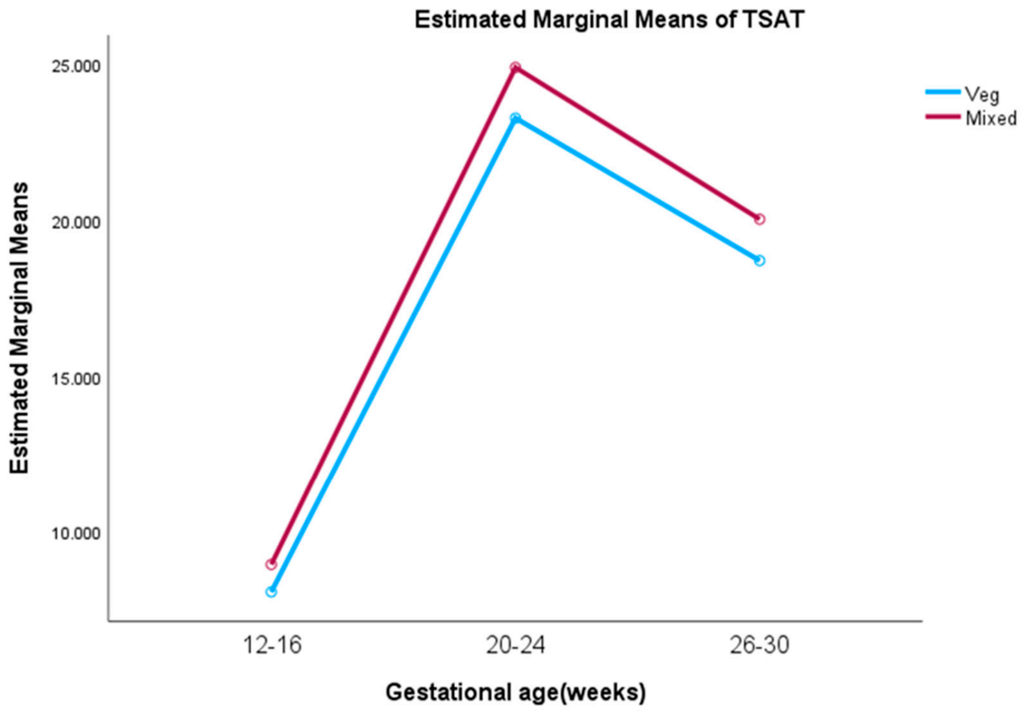

(b)

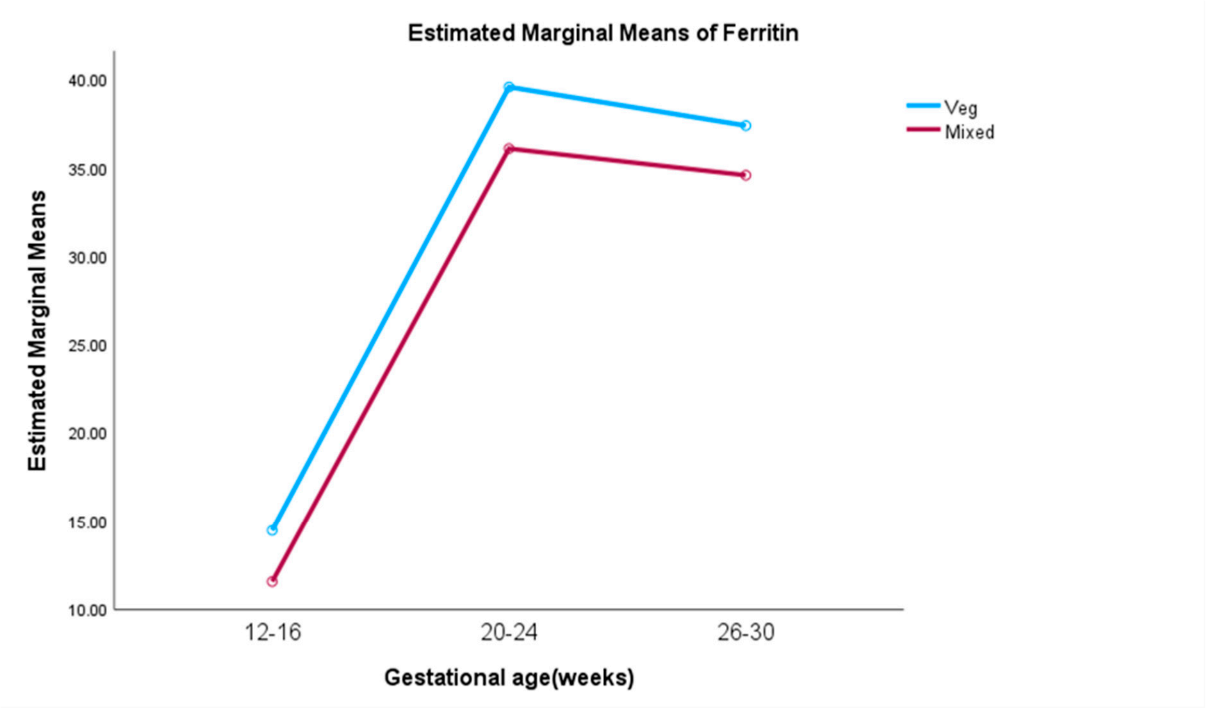
Estimated Marginal Means of Ferritin
Estimated Marginal Means
40.00
35.00
30.00
25.00
20.00
15.00
10.00
12-16
20-24
26-30
Gestational age(weeks)
Veg
Mixed

(c)

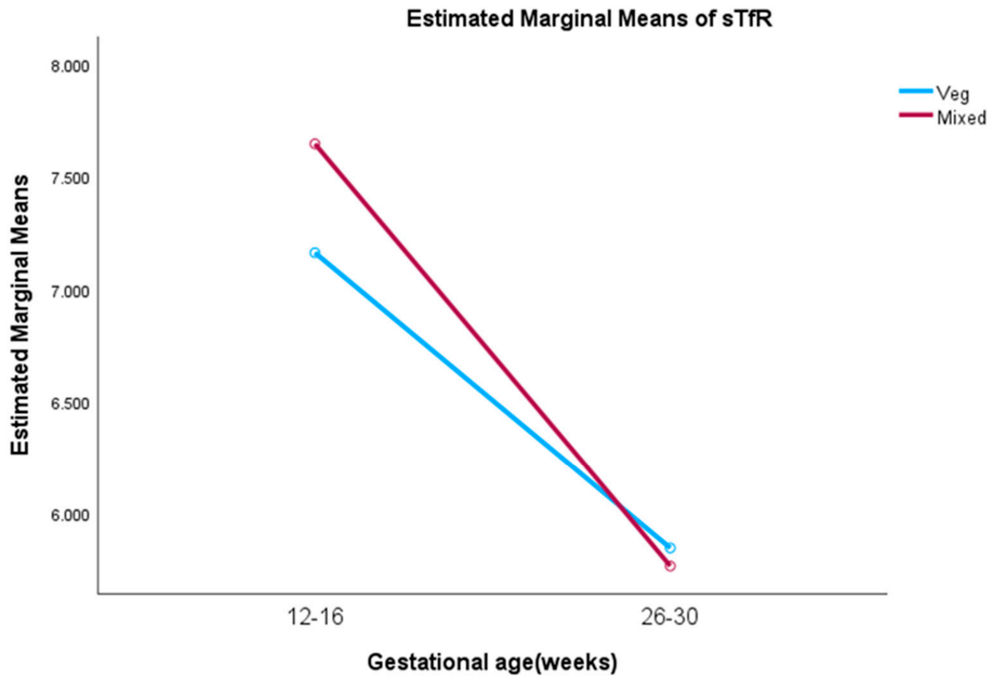
Estimated Marginal Means of sTfR
Estimated Marginal Means
8.000
7.500
7.000
6.500
6.000
12-16
26-30
Gestational age(weeks)
Veg
Mixed

(d)
