# Supplementary material for: Relationship Between Maternal Iron Indices in the Second Trimester with Cord Blood Iron Indices and Pregnancy Outcomes: A Prospective Cohort Study
Source: Nutrients. 2025 May 5;17(9):1584. doi: 10.3390/nu17091584 (PMC12073715; doi:10.3390/nu17091584)
Supplement: Supplementary file 1 [file nutrients-17-01584-s001.zip › Supplementary_Table_S1.pdf]

**Supplementary Table S1.** Correlation between maternal iron indices at each gestational age in the second trimester with cord blood iron indices (n=292).

| **Parameters** | | | **Hb (CB)** | **Ferritin (CB)** | **TSAT (CB)** | **STfR (CB)** |
|---|---|---|---|---|---|---|
| **Hb (M) (g/dL)** | 12-16 GA | ρ | 0.06 | 0.04 | -0.03 | -0.09 |
| | | p-value | 0.25 | 0.44 | 0.55 | 0.32 |
| | 20-24 GA | ρ | 0.15 | -0.02 | -0.20 | 0.10 |
| | | p-value | 0.008 | 0.73 | <0.001 | 0.30 |
| | 26-30 GA | ρ | 0.20 | -0.007 | -0.16 | 0.16 |
| | | p-value | <0.001 | 0.90 | 0.006 | 0.08 |
| **Ferritin (M) (ng/mL)** | 12-16 GA | ρ | 0.07 | -0.07 | 0.04 | -0.007 |
| | | p-value | 0.22 | 0.22 | 0.41 | 0.94 |
| | 20-24 GA | ρ | 0.51 | -0.02 | -0.03 | 0.004 |
| | | p-value | 0.40 | 0.69 | 0.59 | 0.97 |
| | 26-30 GA | ρ | 0.11 | -0.07 | -0.07 | 0.03 |
| | | p-value | 0.06 | 0.18 | 0.22 | 0.74 |
| **TSAT (M) (%)** | 12-16 GA | ρ | 0.01 | -0.07 | 0.09 | -0.12 |
| | | p-value | 0.85 | 0.18 | 0.13 | 0.20 |
| | 20-24 GA | ρ | 0.06 | 0.06 | -0.09 | -0.09 |
| | | p-value | 0.25 | 0.26 | 0.10 | 0.33 |
| | 26-30 GA | ρ | 0.07 | -0.04 | 0.008 | -0.009 |
| | | p-value | 0.21 | 0.41 | 0.89 | 0.92 |
| **sTfR (M) (µg/mL)** | 12-16 GA | ρ | 0.01 | 0.19 | 0.04† | 0.16† |
| | | p-value | 0.90 | 0.04 | 0.67 | 0.08 |
| | 26-30 GA | ρ | 0.05 | 0.05 | 0.08† | 0.05† |
| | | p-value | 0.59 | 0.56 | 0.36 | 0.57 |

M=Maternal; CB=Cord blood; † = Pearson correlation; ρ= rho; GA= Gestational Age.
