# Supplementary material for: Relationship Between Maternal Iron Indices in the Second Trimester with Cord Blood Iron Indices and Pregnancy Outcomes: A Prospective Cohort Study
Source: Nutrients. 2025 May 5;17(9):1584. doi: 10.3390/nu17091584 (PMC12073715; doi:10.3390/nu17091584)
Supplement: Supplementary file 1 [file nutrients-17-01584-s001.zip › Supplementary_Table_S2.pdf]

Supplementary Table S2. Comparison of trends in maternal iron indices (Mean ± SD) across gestational age by maternal diet type (vegetarian vs. mixed).

Maternal Iron Indices			Gestational Age (Weeks)			Interaction F value (p-value)	Within group F statistic	p- value
		n	12-16 GA	20-24 GA	26-30 GA			
Hb (g/dL)	Veg diet	64	9.03 ± 0.73	10.27 ± 0.99	10.56 ± 1.40	1.96(0.15)	41.31	<0.001*
	Mixed diet	228	9.01 ± 0.79	10.42 ± 1.03	10.86 ± 1.21		204.82	<0.001*
TSAT (%)	Veg diet	64	8.07 ± 5.10	23.27 ± 16.46	18.70 ± 14.46	0.04(0.95)	21.89	<0.001*
	Mixed diet	228	8.95 ± 10.43	24.89 ± 19.57	20.03 ± 14.92		85.40	<0.001*
Ferritin (ng/mL)	Veg diet	64	14.41 ± 32.82	39.53 ± 43.05	37.35 ± 47.73	0.01(0.99)	17.34	<0.001*
	Mixed diet	228	11.51 ± 16.31	36.04 ± 36.93	34.54 ± 27.88		60.42	<0.001*
sTfR (µg/mL)	Veg diet	22	7.16 ± 1.51	-	5.84 ± 0.87	1.86(0.18)	12.78	<0.001*
	Mixed diet	83	7.64 ± 1.60	-	5.76 ± 1.12		98.53	<0.001*

*statistically significant
