# Supplementary material for: Relationship Between Maternal Iron Indices in the Second Trimester with Cord Blood Iron Indices and Pregnancy Outcomes: A Prospective Cohort Study
Source: Nutrients. 2025 May 5;17(9):1584. doi: 10.3390/nu17091584 (PMC12073715; doi:10.3390/nu17091584)
Supplement: Supplementary file 1 [file nutrients-17-01584-s001.zip › Supplementary_Table_S3.pdf]

Supplementary Table S3. Comparison of cord blood iron parameters by maternal diet type (vegetarian vs. mixed).

Cord Blood Iron Indices	Veg diet		Mixed diet		p- value
	n	Median(Q_1, Q_3)	n	Median(Q_1, Q_3)	
**Hb (g/dL)**	63	14.99(13.95,16.00)	223	15.18(14.24,16.28)	0.39
**TSAT (%)**	63	57.02(41.93,73.75)	224	59.03(43.16,77.86)	0.19
**Ferritin (ng/mL)**	62	233.00(165.50,369.27)	225	211.90(133.95,311.50)	0.40
**sTfR (μg/mL)**	22	8.15(7.36,9.13)	83	7.89(7.26,8.73)	0.47
